# Coexistence of a novel SETD2-ALK, EML4-ALK double-fusion in an advanced lung adenocarcinoma patient after alectinib resistant and response to immunotherapy combined with chemotherapy: a case report

**DOI:** 10.1007/s12672-023-00654-x

**Published:** 2023-04-13

**Authors:** Lin Zhu, Jing Qin

**Affiliations:** 1 Department of Thoracic Medical Oncology, Zhejiang Cancer Hospital, Institute of Basic Medicine and Cancer (IBMC), Chinese Academy of Sciences, Hangzhou, 310022 Zhejiang China; 2 Zhejiang Key Laboratory of Diagnosis & Treatment Technology on Thoracic oncology (lung and esophagus), Zhejiang Cancer Hospital, Hangzhou, 310022 P.R. China

**Keywords:** Double *ALK* fusion, Case report, Resistance mechanism, Lung adenocarcinoma

## Abstract

The single echinoderm microtubule-associated protein-like 4 (*EML4*) gene and anaplastic lymphoma kinase (*ALK*) gene fusion is the most common variant of *ALK* rearrangements in non-small cell lung cancer (NSCLC). Herein, we firstly report that coexistence of a novel histone methyltransferase (*SETD2*)*-ALK, EML4-ALK* double-fusion is sensitive to alectinib as first-line therapy, and response to immunotherapy combined with chemotherapy after resistant. The patient responded to alectinib as a first-line therapy and achieved progression-free survival (PFS) for 26 months. After resistance, liquid biopsy showed that the reason of drug resistance was the disappearance of *SETD2-ALK* and *EML4-ALK* fusion variants. In addition, chemotherapy combined with immunotherapy subsequently achieved a survival benefit of more than 25 months. Therefore, alectinib may be a viable therapeutic option for NSCLC patients with double *ALK* fusion and immunotherapy combined with chemotherapy may be a viable therapeutic option when double *ALK* fusion loss may be the mechanism of alectinib resistance.

## Introduction

The anaplastic lymphoma kinase (*ALK*) gene fusion accounts for about 3–6% of non-small cell lung cancer (NSCLC), which is the second identified targetable driver gene followed by epidermal growth factor receptor (*EGFR*) mutations in NSCLC [1]. It usually occurs in young, nonsmoking patients with lung adenocarcinoma [2]. At present, according to the National Comprehensive Cancer Network (NCCN) guidelines, the second-generation *ALK*-tyrosine kinase inhibitors (TKIs), alectinib and brigatinib, and the third-generation *ALK*-TKI, lorlatinib, are the preferred first-line treatment recommendations for advanced NSCLC patients with *ALK* gene rearrangements [3]. In clinical practices, patients who simultaneously undergo double *ALK* fusion are rare, and the fused genes are also diverse [4–10]. In this report, we present for the first time an unreported case of lung adenocarcinoma with double *ALK* fusions of echinoderm microtubule-associated protein like 4 (*EML4*) and histone methyltransferase (*SETD2*), which is sensitive to alectinib. When the drug resistance to *AKI*-TKIs occurred, the next-generation sequencing (NGS) test showed that double *ALK* fusions disappeared. Subsequent immunotherapy combined with chemotherapy is effective and achieves long-term survival benefit.

## Case presentation

**Fig. 1:**
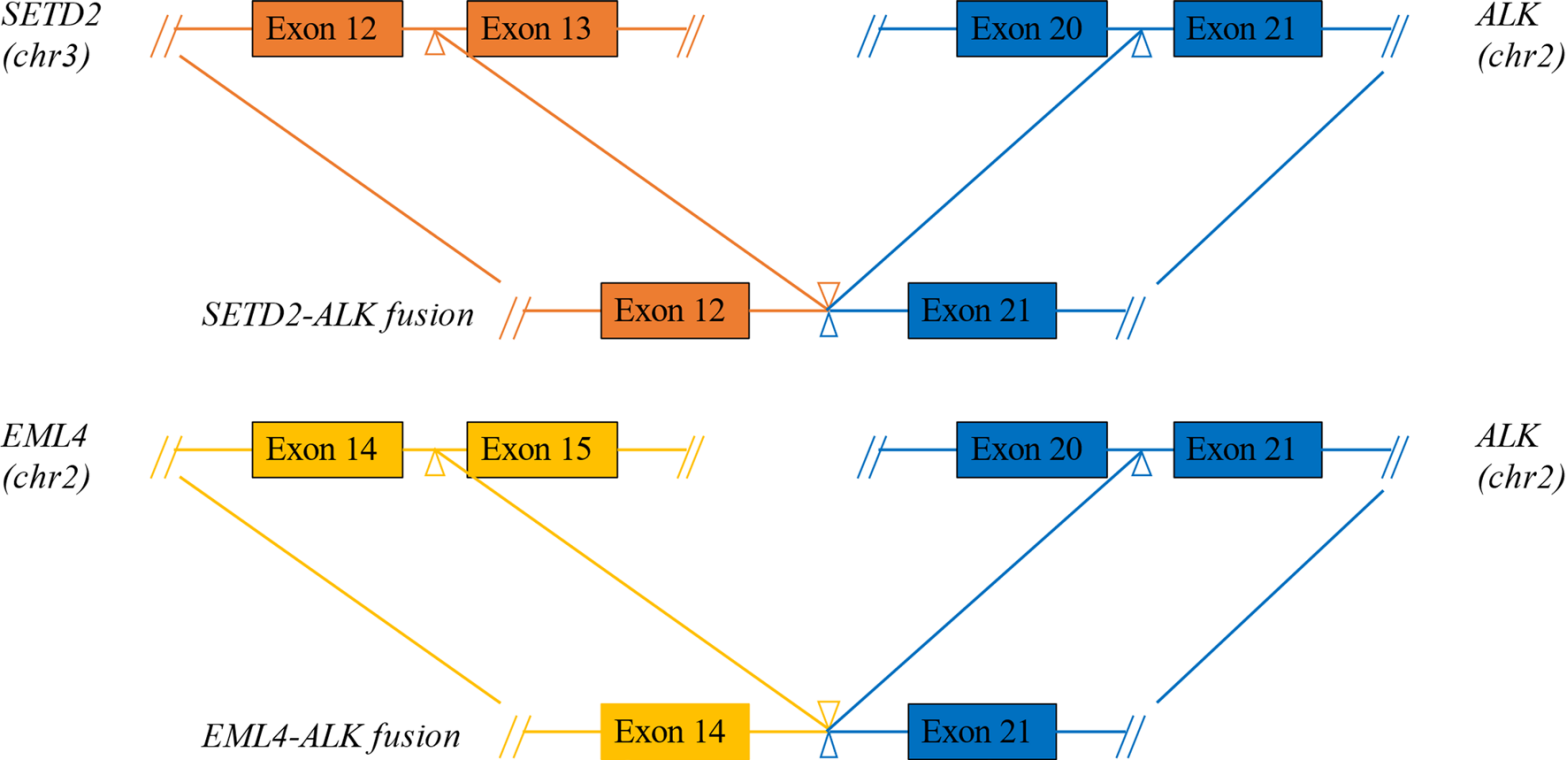
Simulated picture of *EML4-ALK* and *SETD2-ALK* double-fusion

A 28-year-old male with no smoking history presented to the hospital with a persistent cough for half a month in August 2018. Chest computed tomography (CT) scan revealed left upper lobe bronchial stenosis, enlarged multiple mediastinal (2R, 5, 6, 7 zones) and left hilar lymph nodes with multiple nodules in both lungs, left pleural thickening with bilateral pleural and pericardial effusions. And multiple nodular high-density shadows were seen in the left 3rd and 8th ribs, sternum, C1, 7, 11, 12, and the left adnexa of C1. Central type carcinoma of left lung with intrapulmonary, pleural and bone metastasis was considered. Endoscopic biopsy of the left pleural tissue suggested lung adenocarcinoma with ALK-Ventana D5F3, keratin 7, napsin A and thyroid transcription factor-1 (TTF-1) positivity, and adenocarcinoma cells were also found in pleural effusion and pericardial effusion. And *EML4-ALK* (*E14:A21*, allelic frequency: 14.05%) and *SETD2-ALK* (*S12:A21*, allelic frequency: 25.20%) double-fusion (Fig. 1) were found in the pleural tissue by DNA-based NGS, which can capture all exons of 31 genes that are highly related to the occurrence and development of cancer, and important exons and partial introns of 137 genes that are related to individualized treatment or high frequency mutation of lung cancer, and perform high-depth sequencing up to 1000X. (Burning Rock, Guangzhou, China). His disease was diagnosed as left lung adenocarcinoma with left hilar and bilateral mediastinal lymph nodes, bilateral lungs, pleura and bone metastasis (cT4N3M1c, stage IVB).

**Fig. 2:**
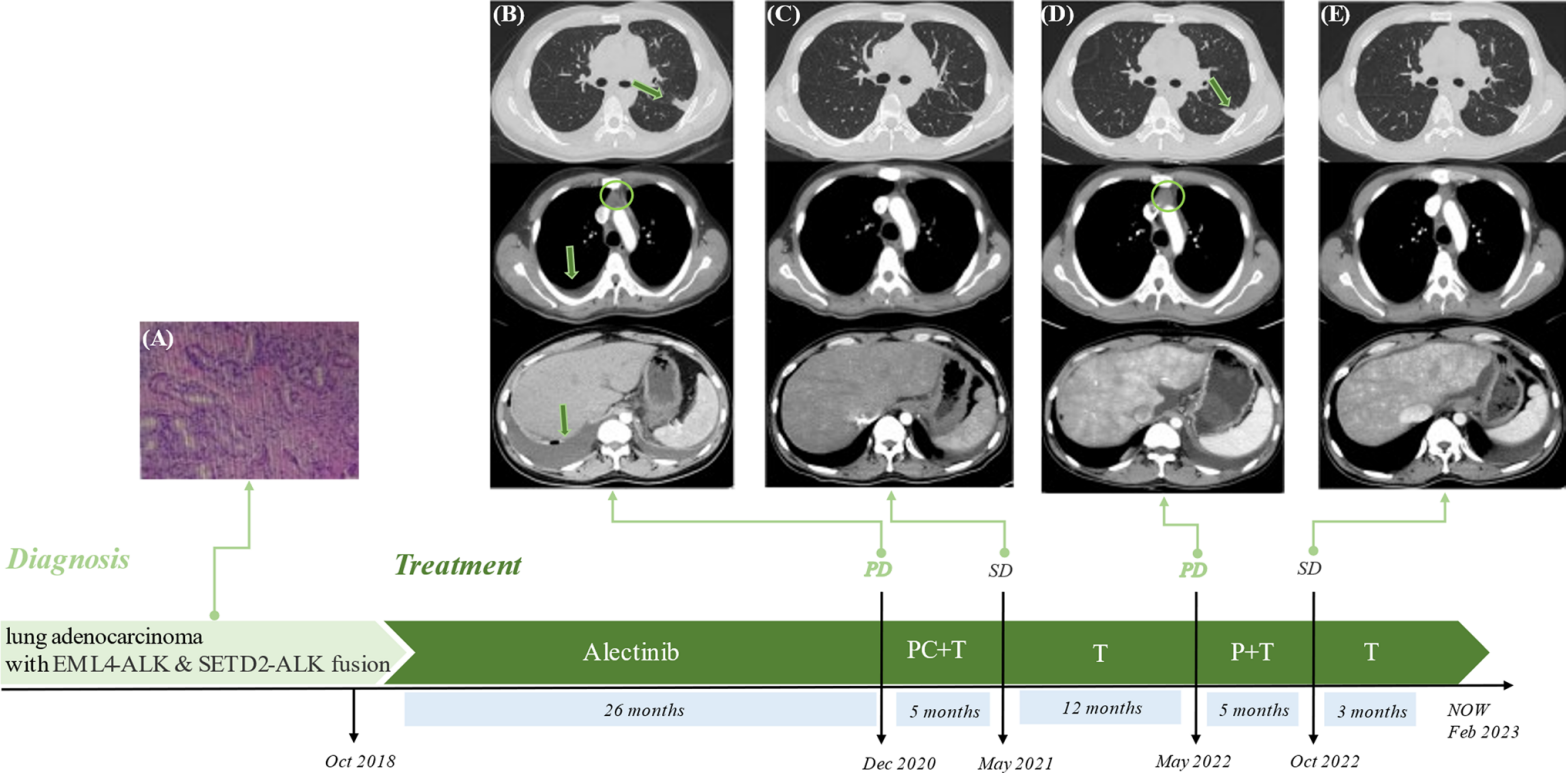
Time axis of diagnosis and treatment

The National Medical Products Administration of China (NMPA) approved alectinib for *ALK*-positive patients with locally advanced or metastatic NSCLC in 2018. The patient began targeted therapy with alectinib (600 mg twice daily) in October 2018. During the period of regular reexamination, the efficacy evaluation was stable disease (SD). After 26 months of treatment, the patient developed chest tightness and shortness of breath again, and chest CT showed bilateral pleural effusion and pericardial effusion again. At the same time, the lesion was enlarged than before, and a large amount of abdominal effusion appeared. No obvious tumor evidence was found in pathological examination of pleural effusion and ascites. According to the patient’s CT and condition, alectinib resistance and disease progression were considered. NGS detection was performed in pleural effusion, abdominal effusion, pericardial effusion and peripheral blood respectively, and no mutation was detected. Given the progress of the disease, pemetrexed plus carboplatin combined with toripalimab was used for six cycles from January 2021, and then maintenance treatment with toripalimab rather than pemetrexed combined toripalimab due to intolerance side effects and willingness of patient. The pulmonary lesion was slightly enlarged again after 16 months of treatment. Considering that pemetrexed had achieved good effect in the past, pemetrexed was added again. After 6 cycles of pemetrexed combined with toripalimab treatment, toripalimab maintenance treatment was performed again. At the deadline of follow-up, the patient continued to receive maintenance treatment with toripalimab. (Fig. 2)

(A) Pathological picture of adenocarcinoma. (B) CT shows disease progression after 26 months of targeted therapy with aletinib; The left interlobar fissure focus and mediastinal lymph node enlargement appeared, together with pleural effusion and new massive intraperitoneal effusion. (C) CT shows that the disease was stable after 6-cycle chemotherapy combined with immunotherapy. (D) CT shows that the left lung lesion and mediastinal lymph node were larger than before after 12 months of immune monotherapy. (E) CT shows that the lesion was stable after another 6-cycle treatment with chemotherapy and immunotherapy.

P, pemetrexed; C, carboplatin; T, Toripalimab; PD, Progressive disease; SD, stable disease.

## Discussion

*ALK* mutations are most commonly caused by fusion with *EML4* [11]. Approximately 61–74% of patients with *EML4-ALK* positive responded to *ALK*-TKIs. There are more than 15 different *EML4-ALK* fusion variants reported in NSCLC. In which *V1 (E13:A20)*, *V2 (E20:A20)* and *V3a/b (E6a/b:A20)* are the most common. A previous retrospective study has found that variant *V1/2* is more sensitive to *ALK*-TKIs than variant *V3a/b*, suggesting that different *EML4-ALK* variants may show different sensitivity to *ALK*-TKIs [12–14]. However, the sensitivity of fusion of “*E14:A21*” in this case has not been previously reported.

*SETD2-ALK* gene fusion is a rare *ALK* fusion, and it has been rarely reported in the previous studies. J. Kang et al. found that *SETD2-ALK* gene fusion at the DNA level expressed the *EML4-ALK* transcripts during mRNA maturation. It plays a role of “mediation”. In addition, the study also found that there was no significant difference in overall survival (OS) between different *EML4-ALK* fusion subtypes (P = 0.386), while multiple *ALK* fusion was significantly higher than that of *EML4-ALK* fusion (P = 0.01). It is possible that tumors with multiple *ALK* fusions are likely to be more addicted to the *ALK* signaling pathway, thus making the *ALK*-TKIs have more profound effects [14]. However, in this case, the patient with *EML4-ALK* and *SETD2-ALK* double-fusion had a progression-free survival (PFS) of 26 months after targeted therapy with alectinib, which was similar to the median PFS of 25.7 months assessed by an independent evaluator in the ALEX trial [15]. Therefore, NSCLC patients with double or compound mutations still can have response and survival benefit to targeted therapies. *SETD2*-*ALK* can be a rare fusion mutation which is sensitive to the treatment of *ALK* inhibitors.

Individualized treatment should be given according to the mechanism of *ALK*-TKIs resistance. 56% of patients with *ALK* gene fusion mutations developed *ALK* resistance mutations after the use of second-generation *ALK* inhibitors, with *ALK G1202R* becoming the most common *ALK* resistance mutation. The third generation *ALK*-TKIs, lorlatinib, can effectively inhibit the *ALK* resistance mutation. But in the remaining 44%, the mutation disappeared after the use of second-generation *ALK* inhibitors [16]. The ALUR study showed that for patients who failed to detect *ALK* fusion after first-line crizotinib resistance, compared with patients with *ALK* fusion after treatment, the objective response rate (ORR) of second-line treatment with alectinib was lower, and there was no significant improvement compared with chemotherapy. The results suggest that it may be more reasonable to choose the treatment mode of chemotherapy when *ALK* fusion is not detected after *ALK*-TKIs resistance [17]. Furthermore, a retrospective study including 20 patients with *ALK* fusion and disease progression after first-line aletinib treatment showed that the curative effect of *ALK*-TKIs in patients with secondary mutation of *ALK* is better than that in patients without secondary mutation of *ALK* (PFS: 242d vs. 75d, P = 0.05, HR = 0.46, 95% CI: 0.18–1.2). For patients without mutation, most of the them received at least first-line chemotherapy, and it was found that chemotherapy achieved better PFS (168d vs. 75d, P = 0.035, HR = 0.47, 95% CI: 0.19–1.2) than *ALK*-TKIs [18]. Therefore, the benefit of using *ALK*-TKIs is limited for patients who have *ALK* fusion before treatment but whose *ALK* fusion disappears after treatment, and the benefit may be more obvious when chemotherapy is used for subsequent treatment compared with targeted therapy.

In conclusion, this is the first report of an advanced and metastatic lung adenocarcinoma patient harbored *EML4-ALK* and *SETD2-ALK* double-fusion variation and successively response to alectinib and immunotherapy combined with chemotherapy after alectinib resistant. Loss of both *EML4-ALK* and *SETD2-ALK* double-fusion may be the mechanism of aletinib resistance for this patient. Immunotherapy combined with chemotherapy as second-line treatment may be a viable therapeutic option for NSCLC patients with double-*ALK* fusion when loss of double-fusion is the mechanism of *ALK*-TKIs resistance. However, the study also has certain limitations that must be acknowledged. During the progress of targeted treatment with aletinib, cancer cells were not found in pleural effusion, peritoneal effusion and pericardial effusion, so NSG detection of serous effusion may be negative. At the same time, the detection rate of plasma NGS detection was lower than tissues, which may affect the accuracy of NGS detection results. The consideration of *ALK* gene loss was more from our understanding of the disease, and there was no definite basis for it. Readers should interpret it with caution.

## Data Availability

All data generated or analysed during this study are included in this published article.
